# Ten Simple Rules for Getting Ahead as a Computational Biologist in Academia

**DOI:** 10.1371/journal.pcbi.1002001

**Published:** 2011-01-06

**Authors:** Philip E. Bourne

**Affiliations:** Skaggs School of Pharmacy and Pharmaceutical Science, University of California San Diego, La Jolla, California, United States of America

Getting a promotion or a new position are important parts of the scientific career process. Ironically, a committee whose membership has limited ability to truly judge your scholarly standing is often charged with making these decisions. Here are ten simple rules from my own experiences, in both getting promoted and serving on such committees, for how you might maximize your chances of getting ahead under such circumstances. The rules focus on what might be added to a CV, research statement, personal statement, or cover letter, depending on the format of the requested promotion materials. In part, the rules suggest that you educate the committee members, who have a range of expertise, on what they should find important in the promotion application provided by a computational biologist. Further, while some rules are generally applicable, the focus here is on promotion in an academic setting. Having said that, in such a setting teaching and community service are obviously important, but barely touched upon here. Rather, the focus is on how to maximize the appreciation of your research-related activities. As a final thought before we get started on the rules, this is not just about you, but an opportunity to educate a broad committee on what is important in our field. Use that opportunity well, for it will serve future generations of computational biologists.

## Rule 1: Emphasize Publication Impact, Not Journal Impact

Reviewers who do not know your work well, unless told otherwise, will often judge that work primarily by the journals in which it appears. If the majority of your papers are in *Nature* and *Science*, then let the system continue to fool the reviewer. For the rest of us, it is important to emphasize that the impact of the journal does not necessarily reflect the impact of your paper. Include any data that reflect the value of your work regardless of the journal. The number of times the paper has been cited and the download statistics for that paper are obvious metrics, but should be put in context. A few citations and downloads do not necessarily mean the paper is not valuable in a narrow field. Tell the committee why it has significant impact in that field. There are also other less likely sources of support that can help. Coverage by the Faculty of 1000, press releases, blogs, and any positive commentary on the paper by others are also valuable indicators of impact.

## Rule 2: Quantify and Convince

Reviewers may not be that familiar with the concept of article-level metrics and what they say about your science—where applicable, convince them in your application. Let me use an example. The very first article I wrote in this series was titled “Ten Simple Rules for Getting Published” [1]. It has been downloaded over 65,000 times, which is about 35 times per day since it was published 5 years ago. At the same time, according to Google Scholar it has been cited 30 times and according to ISI Web of Knowledge 11 times. The implication is that it has had some scholarly impact that is not reflected by the more traditional citation metric. In this case, the scholarly impact is mainly pedagogical in that it assists in professional development. This is easily overlooked by a promotion committee, but of some value in academic promotion. Metrics may not tell the whole story, for instance, in work that is relatively new. Use your application to inform the reviewers why you believe your work is significant.

## Rule 3: Make Methods and Software Count

Keep statistics on software and methods use. For example, keep statistics on the number and diversity of users of the software, publications that cite the software, and the impact of those citations. For software that is modular, include the diversity of applications to which those methods and/or software have been applied. Describe what it took to develop the methods and/or software and what impact that has on the community. Many reviewers will not appreciate what it takes to develop and maintain methods and/or software for the community. Do what you can to help the reviewer with details of your time and resources, and that of others, in maintaining the software for the good of the community. Educate the committee on what open source implies, assuming your software is open source. Indicate as best you can how your efforts in software and methods bring credit to the institution.

## Rule 4: Make Web Sites Count

This follows from Rule 3, but applies specifically to Web sites where Google Analytics, AWStats, and other tools can be used to quantify the impact your work has had and present those statistics to reviewers. Another irony is that papers about Web sites are rarely read, but they are highly cited if your resource is useful. Hence, they can be used to enhance your standing. Good professional conduct should dictate that you only write such papers when you have something substantively new to report regarding improvements to the Web site. Spreading citations over multiple papers just to enhance your H-factor while not adding anything substantively new speaks poorly of you and to the value system we use to evaluate scholars.

## Rule 5: Make Data Deposition, Curation, and Other Related Activities Count

Maintain records on your data-related activities, namely public accessibility, how much curation and other effort went into providing these data, and how much these data are used. Currently, there is no way to quantify the impact your public contributions of data have had on science; therefore, try to ensure that such contributions have an associated publication. Contact data resources to see if they can provide metrics for how frequently data you have contributed has been accessed and include that information in your list of accomplishments.

## Rule 6: Use Modern Tools to Emphasize/Quantify Your Academic Standing

Increasingly, tools are available to impart to reviewers your scholarly standing. For example, ResearcherID from Thomson Reuters [2] will provide graphs on the total number of citations per year, average number of citations per article, and so on. However, these are only for publications found in ISI databases, which can be limited for a multidisciplinary researcher. PubNet [3] will provide your collaborative network from PubMed where each node on the network is a researcher you have published with and the thickness of edges reflects the number of times you have published together. BioMedExperts [4] provides similar data. Again, this can be somewhat limiting for multidisciplinary researchers. Bolster these statistics by indicating the full range of your scholarly activities not covered by the tools. Adding papers manually to the tracking resource can often help as well.

## Rule 7: Make an Easily Digestible Quantified Summary of Your Accomplishments

Reviewers are often faced with many applications for promotion to review, and your accomplishments are easily lost in a long CV. This is particularly true if the reviewer is trying to sort out what you have accomplished in a specific time frame, as would often be the case when considering a promotion. One way to summarize accomplishments is as a bulleted list in a cover letter or some other allowable personal statement. Items on that list should include, where appropriate: published and accepted papers, pending and funded grants, including the amount coming to your institution, summarized accomplishments in software, data, and methods as per Rules 3, 4, and 5, students mentored and in what capacity, courses offered and their standing, other educational and outreach activities, company involvement, professional activities (e.g., editorial boards, scientific advisory boards), invited lectures, and awards. The idea is not to provide details here—your CV should do that—just numbers for easy and quick comprehension.

## Rule 8: Make the Reviewers’ Job Easy

Often, one or more of the reviewers looking at your application are going to be responsible for writing a summary of why, or why not, your advancement was granted. Again, unless the reviewers are very familiar with your work they will appreciate a candid, quantitative and honest discussion of your accomplishments. But take heed of Rule 10. Where such a discussion should be included depends on the form of your application—usually as a cover letter or part of your personal statement is appropriate. Whatever the form, it should be brief and highlight, in a way that can be understood by a non-expert, what was done and why it is of high impact and, if available, how others have followed up on the accomplishments. These highlights should be peppered with citations and quantitative data that a reviewer can easily reference should they choose to do so. More often than not the reviewer will appreciate this summation and it will be reflected in the letter they write.

## Rule 9: Make the Job of Your References Easy

Often your application will include letters of support from external references, some chosen by you, others chosen by the reviewers. For the ones you choose, send those references the same summary you provide the reviewers (Rule 8). The reviewers will likely know your work well, which is why they were chosen. Notwithstanding, a good factual summary can help in their writing a reference letter, which is a significant undertaking when done well. They will thank you for it. You might even include information they would appreciate, that the committee would not—for example, specific details of research if you and the reviewer are in the same field.

## Rule 10: Do Not Oversell Yourself

This may be obvious, but have an impartial third party look over your application and have them give you a candid opinion; perhaps a senior member of your institution not on your committee. Don’t oversell yourself with flowery adjectives. Show, don’t tell; that means, enumerate facts. If you head a laboratory, even though it is your file under consideration, it is really the work of the collective you are highlighting—be clear and fair about that. Just state the facts—if you have done well, you will do well. It is as simple as that.

I have placed significant emphasis on what to include in a cover letter or personal statement that accompanies your CV, research statement, and perhaps other materials, such as teaching evaluations. I have not discussed preparing a good CV since such information is available on the Internet and elsewhere already. What has not been covered before, as far as I am aware, is how a computational biologist in academia might maximize their chances of being promoted by a committee that is not fully appreciative of the field.

As always, we welcome your comments. I would particularly like to hear additional/alternative advice from those like myself who have been through this process a number of times. In closing, I can only offer an example of such materials that I think helped me get promoted last time around (see Text S1).

## Supporting Information

Text S1 Example support letter.
(PDF)
