# Supplementary material for: Ten Simple Rules for Getting Ahead as a Computational Biologist in Academia
Source: PLoS Comput Biol. 2011 Jan 6;7(1):e1002001. doi: 10.1371/journal.pcbi.1002001 (PMC3017106; doi:10.1371/journal.pcbi.1002001)
Supplement: Text S1 — Example support letter. (PDF) [file pcbi.1002001.s001.pdf]

UNIVERSITY OF CALIFORNIA, SAN DIEGO UCSD

BERKELEY • DAVIS • IRVINE • LOS ANGELES • MERCED • RIVERSIDE • SAN DIEGO • SAN FRANCISCO SANTA BARBARA • SANTA CRUZ

SKAGGS SCHOOL OF PHARMACY AND PHARMACEUTICAL SCIENCES

March 2009

To Whom it May Concern:

The following defines the accomplishments of our laboratory for the calendar years 2006, 7, and 8:

- Published 34 peer reviewed papers (5 additional pending).
- Published an additional 11 Editorials including a professional development series.
- Currently hold grants worth $17.8 million.
- Won the Protein Data Bank (PDB) 5 year renewal starting Jan. 1, 2009 – A $9.6M award from the National Science Foundation.
- Developed and supported web sites used by over 220,000 scientists every month who collectively download data equivalent to one quarter of the US Library of Congress.
- Saw the journal PLoS Computational Biology that we co-founded achieve number one ranking in the field of computational biology with an impact factor of 6.
- Co-Founded SciVee Inc., filed a patent providing unique IP to the company, won a UCSD entrepreneurial award and obtained STTR funding to support the business.
- Co-edited and submitted a Second Edition of the successful reference book Structural Bioinformatics to Wiley-Blackwell.
- Began a new course in professional development for BMS and other graduate students while continuing two other full courses.
- Delivered 31 invited lectures worldwide.
- Graduated 5 PhD and 5 Masters students (through York University, UK).

More detail is provided on a few of these points.

<u>Research</u>

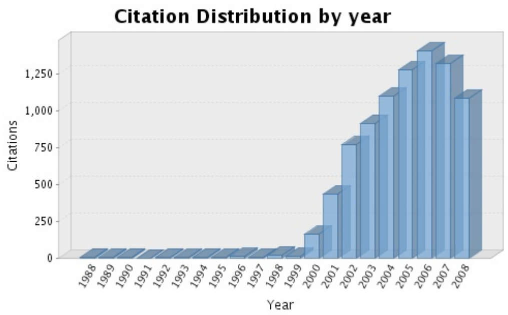

Our work has consistently achieved over 1,250 citations per year (see Fig. source Thomson Reuters). While the majority of citations come from the data resources maintained by the laboratory, we have impacted five distinct fields in the past three years with fundamental contributions as follows.

1. **Evolution** – in the prior period we established protein structure as a fundamental tool in the study of evolution (*PNAS* 2005 102(2): 373-378; 55 citations) by reconstructing the tree of life from protein domain content. In this period, among other discoveries, we established a link between how the changing metal ion concentrations of the ocean over geological time scales influenced protein structure and hence function (*PNAS* 2006 103(47) 17822-17827). In a separate study (*J. Mol. Evol.* 2008 66(5) 494-

504) we discovered two new types of proteosome which are currently being studied structurally by Robert Huber's laboratory and finally we discovered a novel protein kinase (*PLoS ONE*, 3(2): e1597).

2. **Drug Discovery** – in a reverse engineering of the drug discovery process we are able to routinely determine alternate targets for major pharmaceuticals (*BMC Bioinformatics*, 2007, 8(Suppl 4):S9; *PNAS*, 2008, 105(14) 5441-5446). Using this process we were able to offer an off-target receptor, SERCA, for select estrogen receptor modulators (e.g., tamoxifen) which explains the side effects seem in using these drugs (*PLoS Comp. Biol.*, 3(11) e217), a finding highlighted by NIGMS. Intriguing stories on repositioning Parkinson's disease drugs to treat TB and the cause of failure of the Pfizer drug Torcetrapib will be published in the next month or two with many more stories to follow.

3. **Scholarly Communication** – represents a completely new area of study in our laboratory based upon our work in open access publishing. We have pioneered ways to enhance traditional learning by integrating printed materials with video (*CT Watch*, 2007 3(3) 26-31) and by semantically enriching the content of Pubmed Central (*NAR* 36(S2) W385-389. Finally in collaboration with Microsoft we have developed advanced authoring tools to be used by the Public Library of Science and other publishers.
4. **Immunology** – Aside from contributions to the Immune Epitope Database (*NAR*. 2008, 36(S2) W513-W518; *BMC Bioinformatics*, 2007, 7(1), 341) which is attempting to catalogue all reported B and T cell epitopes we have established a benchmark to measure the ability to predict epitopes and continue to address the important problem of epitope prediction (*BMC Structural Biology*, 2008, 7(1):64).
5. **Allostery, protein-protein interactions, protein classification** – remain areas in which we continue to have impact with at least half a dozen new papers.

<u>Teaching</u>
We have continued to direct and teach two courses. Pharm201 (Biological Data and Analysis Tools) in the fall quarter is one of the four core courses in the Bioinformatics graduate program and I teach 85% of the lectures. SPPS205 (Pharmacy Informatics) is a required course in the spring quarter for all first year PharmD students and I teach 20% of the lectures. In winter quarter of 2008 I introduced a professional development course in which I taught 90% of the lectures. I will modify and continue this course in 2009. I also gave guest lectures in Med260, Biom202 and Pharm202.

<u>University/Public</u>

We continue to provide a broad array of public service activities; we wish to highlight one important development over the past three years. Through work as co-Founder and Editor-in-Chief of the open access journal *PLoS Computational Biology* (impact factor 6) we have come to realize the promise of new ways to communicate science at all levels. Beyond free access to everyone, the availability of the full text of research articles on-line offers opportunities for new knowledge discovery across the literature and for improving our ability to comprehend the literature. In the past three years we have established a scholarly communications group (currently 7 people) in the Engineering School to pioneer new modes of scientific communication (*Nuc. Acids Res. 2008* 36(S2) W385-389; *Pacific Symposium on Biocomputing*, 2008, 640-651). It became clear that sustaining such an effort could not be done on federal funding, but required a business model. To this end we co-founded SciVee Inc. which has received an STTR grant, won an entrepreneurial award for new innovation, has an SBIR pending and has a CEO seeking funding from traditional publishers to develop these ideas.

We hope you find this summary useful.
